# Ensemble learning-based radiomics with multi-sequence magnetic resonance imaging for benign and malignant soft tissue tumor differentiation

**DOI:** 10.1371/journal.pone.0286417

**Published:** 2023-05-31

**Authors:** Seungeun Lee, So-Yeon Lee, Joon-Yong Jung, Yoonho Nam, Hyeon Jun Jeon, Chan-Kwon Jung, Seung-Han Shin, Yang-Guk Chung

**Affiliations:** 1 Department of Radiology, Seoul St. Mary’s Hospital, College of Medicine, The Catholic University of Korea, Seoul, Republic of Korea; 2 Division of Biomedical Engineering, Hankuk University of Foreign Studies, Seoul, Gyeonggi‐do, Republic of Korea; 3 Department of Pathology, Seoul St. Mary’s Hospital, College of Medicine, The Catholic University of Korea, Seoul, Republic of Korea; 4 Department of Orthopedic Surgery, Seoul St. Mary’s Hospital, College of Medicine, The Catholic University of Korea, Seoul, Republic of Korea; Tecnologico de Monterrey, MEXICO

## Abstract

Many previous studies focused on differentiating between benign and malignant soft tissue tumors using radiomics model based on various magnetic resonance imaging (MRI) sequences, but it is still unclear how to set up the input radiomic features from multiple MRI sequences. Here, we evaluated two types of radiomics models generated using different feature incorporation strategies. In order to differentiate between benign and malignant soft tissue tumors (STTs), we compared the diagnostic performance of an ensemble of random forest (R) models with single-sequence MRI inputs to R models with pooled multi-sequence MRI inputs. One-hundred twenty-five STT patients with preoperative MRI were retrospectively included and consisted of training (n = 100) and test (n = 25) sets. MRI included T1-weighted (T1-WI), T2-weighted (T2-WI), contrast-enhanced (CE)-T1-WI, diffusion-weighted images (DWIs, b = 800 sec/mm^2^) and apparent diffusion coefficient (ADC) maps. After tumor segmentation on each sequence, 100 original radiomic features were extracted from each sequence image and divided into three-feature sets: T features from T1- and T2-WI, CE features from CE-T1-WI, and D features from DWI and ADC maps. Four radiomics models were built using Lasso and R with four combinations of three-feature sets as inputs: T features (R-T), T+CE features (R-C), T+D features (R-D), and T+CE+D features (R-A) (Type-1 model). An ensemble model was built by soft voting of five, single-sequence-based R models (Type-2 model). AUC, sensitivity, specificity, and accuracy of each model was calculated with five-fold cross validation. In Type-1 model, AUC, sensitivity, specificity, and accuracy were 0.752, 71.8%, 61.1%, and 67.2% in R-T; 0.756, 76.1%, 70.4%, and 73.6% in R-C; 0.750, 77.5%, 63.0%, and 71.2% in R-D; and 0.749, 74.6%, 61.1%, and 68.8% R-A models, respectively. AUC, sensitivity, specificity, and accuracy of Type-2 model were 0.774, 76.1%, 68.5%, and 72.8%. In conclusion, an ensemble method is beneficial to incorporate features from multi-sequence MRI and showed diagnostic robustness for differentiating malignant STTs.

## Introduction

Soft tissue tumors (STTs) are heterogeneous groups of tumors with different cellular origin and heterogeneous tissue components [1]. As there are more than 50 histologic subtypes in STTs, various extracellular components such as hemorrhage, mineralization, fat, and myxoid tissue are mixed in a mass with diverse distribution [2]. These components show distinctive signal intensity on a series of magnetic resonance images (MRI) and radiologists identify the diagnosis through the correlated signal intensity of certain components. Standard interpretation of STTs is based on a conventional MR sequence of T1- and T2-weighted images (T1-WI, T2-WI) and contrast-enhanced (CE)-T1-WI if contrast media is available. STTs with larger size, lobular contours, and heterogeneous components on conventional MRI are more likely to be malignant tumors, but there is no definite diagnostic qualitative criteria for diagnosis of soft tissue sarcoma because diverse subtype of soft tissue sarcoma can show various imaging findings [3–5]. Recently, advanced imaging techniques such as diffusion-weighted imaging (DWI), dynamic contrast-enhanced imaging, and MR spectroscopy are combined to acquire functional information including cellularity, vascularity, or metabolism [6]. This additional information could support radiologists’ assessment of conventional sequences when differentiating between benign and malignant tumors. However, it is still challenging to diagnose benign or malignant soft tissue tumor based on imaging alone, because multi-sequence MRI could lead to confusion by providing discrepant findings with benign or malignant histology of tumor itself, like low apparent diffusion coefficient (ADC) value of epidermal inclusion cyst, high ADC value of sarcoma with myxoid matrix or high-flow hemodynamic profile on dynamic contrast-enhanced imaging of vascular anomaly [7–9].

Radiomics is an approach utilizing high-dimensional quantitative features extracted from images to build a classification model. Recently, many radiomics studies have been published to distinguish the malignancy and histologic grades of STTs using conventional MRI [10–15]. In early studies, features from two MR sequences (T1-WI and T2-WI with or without fat suppression) were pooled in a single set of combined MR features and used as input for classification models [11, 12, 16]. More recent studies have developed separate models from each of the two MR sequences (fat-suppressed T2-WI and T1-WI with or without CE) and models based on combined features from two MR sequences [13, 14, 17]. They also pooled all MR features into a single set to construct a combined features set with the same method as in previous studies. When differentiating soft tissue sarcoma from benign tumors, diagnostic performance is higher when multiple MRI sequences are reviewed together [1, 18]. Therefore, radiomics models from multiple MR sequences including CE images and DWIs may be more useful than radiomics models from two conventional MR sequences. However, traditional pooling methods have possibility of loss of information from each MR sequences, which can result in decreased generality to histologically diverse tumors. Therefore, we assumed that the ensemble model was suitable for creating a model that reflects all information in each sequence and aimed to verify it.

The ensemble learning approach is a combination method of multiple classifiers to achieve better performance than single-model classification [19]. Predictions from several different classification models are used as input for the ensemble model and final predictions are yield by proportion of votes to favor one class. This process can resolve the reproducibility problem by eliminating selection bias of single-classifier algorithms [20]. We assumed that an ensemble learning of several different classification models which are respectively derived with input of a single MR sequence, would perform better by preserving information from different sequences. It can also be similar to the interpretation process by radiologists, by the point that they interpret the image finding by combining information from conventional and advanced MR sequences, and their decisions based on multi-sequences are usually more accurate than judging based on single-sequence alone. In field of radiomics study, it is not well understood if it is more accurate to combine the classification results from multiple MR sequences. In addition, no definite method to combine radiomic features has been established or evaluated.

In this study, we aimed to evaluate diagnostic performance of an ensemble of random forest (R) models with single-sequence inputs compared to R models with multi-sequence inputs for differentiating benign and malignant soft tissue tumors

## Materials and methods

This retrospective study was approved by our institutional review board, and the requirement for informed consent was waived. This work was supported by the National Research Foundation of Korea grant funded by the Korea government (Ministry of Science and ICT) 2022R1A2C4002395.

### Patient population

**Fig 1:**
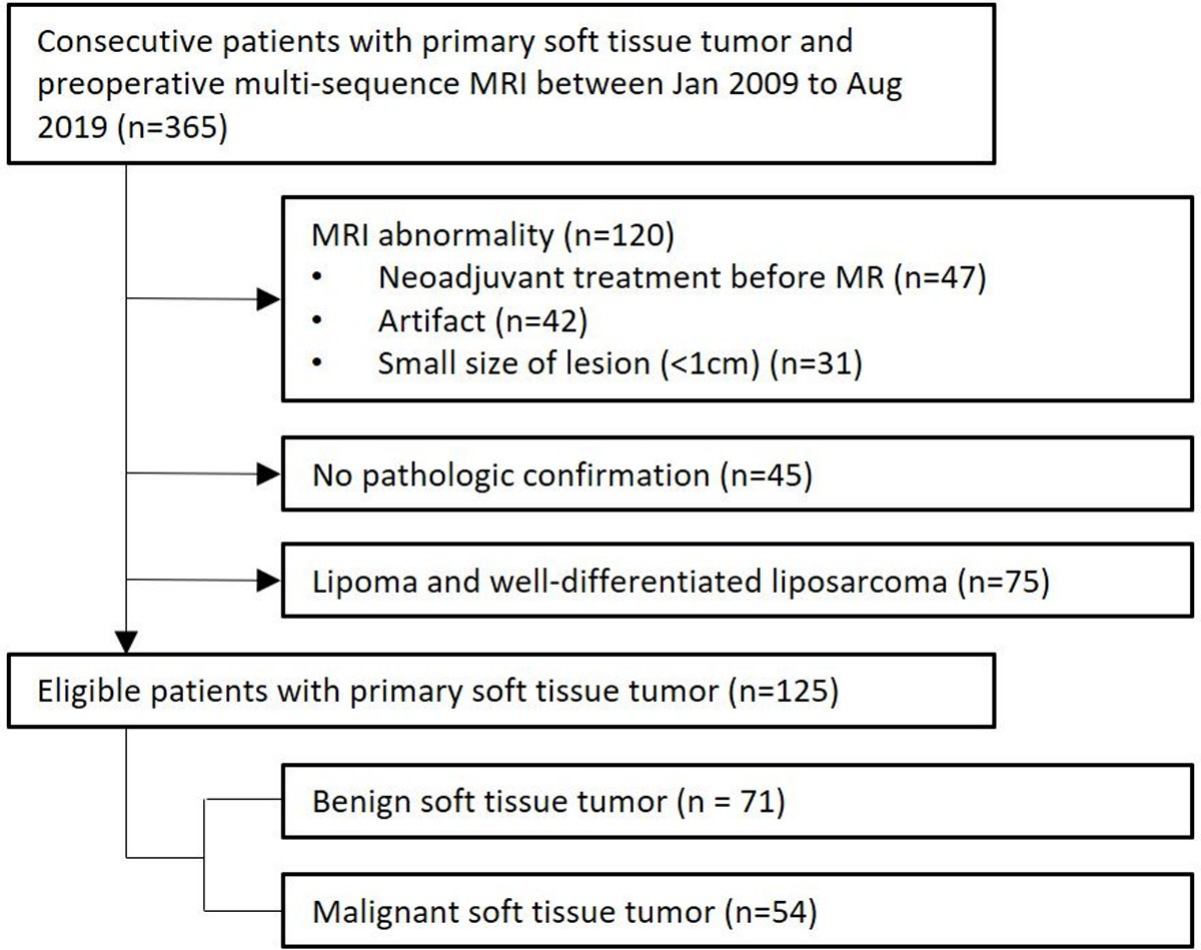
Flowchart of patient inclusion.

From January 2009 to August 2019, a total of 365 patients at our institution underwent 3.0T preoperative multi-sequence MRI including T1-WI, T2-WI, CE-T1-WI, DWIs, and ADC map for primary STT evaluation. The cohort was reduced to 125 patients according to the following exclusion criteria (Fig 1). First, multi-sequence MRI was performed after any neoadjuvant treatment was excluded (n = 47). Tumors showing distortion on MR images due to artifacts (n = 42) and lesions less than 1 cm in size (n = 31) were excluded. Second, all tumors required pathology confirmed by surgical excision with histological analysis on the excisional sample performed by one pathologist (C.K.J., 15 years of experience in musculoskeletal tumors) and patients without pathologic confirmation were excluded (n = 45). Finally, well-differentiated adipocytic tumors including lipomas and well-differentiated liposarcomas were excluded (n = 75) because of abnormal signal intensity on DWI and ADC map as a fat-suppressed sequence [21]. All included patients were previously involved in the patient cohort of our previous studies [22]. The previous studies utilized diffusion-weighted images for differentiation of benign and malignant STT and T2-weighted Dixon sequence for soft tissue sarcoma margin evaluation, respectively.

### MRI protocols

MR images were acquired before surgery or neoadjuvant treatment in all patients. MRI was performed using 3.0T scanner (MAGNETOM Verio; Siemens Healthineers, Erlangen, Germany) with dedicated surface coils depending on tumor location. The standard MRI protocols included the longitudinal fat-suppressed T2-weighted turbo spin-echo (TSE) sequence, axial T1-weighted TSE sequence, axial T2-weighted TSE sequences with and without fat suppression, and longitudinal and axial fat-suppressed contrast-enhanced T1-weighted TSE sequence. MRI parameters are shown in S1 Table. Before contrast enhancement, a single-shot spin-echo echo-planar DWI sequence was also obtained on the axial plane. A parallel imaging technique using GeneRalized Autocalibrating Partially Parallel Acquisitions was combined with an acceleration factor of two. Sensitizing diffusion gradients were applied with b values of 0 and 800 sec/mm^2^ sequentially in the x, y, and z directions [23]. Pixel-based ADC maps were created from DWIs based on mono-exponential calculation using commercial software and a workstation (Leonardo MR Workplace; Siemens Medical Solution, Erlangen, Germany). For contrast-enhanced image, the contrast agent was injected with 0.1 mmol per kg body weight gadobutrol (Gadovist; Bayer, Berlin, Germany). To ensure a stable injection speed, the contrast agent was injected intravenously with an automatic injector (Spectris Solaris; Medrad, Indianola, Pennsylvania) at a rate of 2 mL/s. Subsequently, 20 mL of physiological saline was injected at the same rate.

### Radiomics model acquisition

#### 1. Tissue segmentation

Segmentation was initially performed by one radiologist (S.L, 3 years of experience on musculoskeletal radiology) using the semiautomatic region intensity filter method, which was implemented using ITK-SNAP software, version 3.8.0 (open source, http://www.itksnap.org/) [24] on each MR sequence image and manually revised. Volume of interest (VOI) was drawn along the entire mass except for the most peripheral portions to avoid partial-volume effects. To review the reproducibility of VOI segmentation, final correction of the peripheral portion in VOI was edited in 20 tumor images by three readers: one student (H.J.J.) and two radiologists (S.L., S.Y.L., 3 and 13 years of experience in musculoskeletal radiology, respectively).

#### 2. Image preprocessing and radiomic feature extraction

The single VOI was selected by consensus of the three readers (H.J.J., S.L., and S.Y.L.) for further preprocessing steps. After VOI confirmation, normalization of the all 5 image sequences were conducted with the following equation: $(x)=\frac{s\left(x–\mu_{x}\right)}{\sigma_{x}}*1000$, with *f* (*x*) as normalized intensity, *x* as original intensity, *μ*_*x*_ as mean, and *σ*_*x*_ as standard deviation of image signal intensity [25]. As the average ranges of normalized signal intensity in all 5 image sequences extend from 5166 to 9704, gray-level discretization was done with a bin count of 200 to acquire the adequate contrast level and noise reduction in result image. Finally, the VOIs were resampled at a spatial resolution of 1 × 1 × 1 mm^3^ in T1-WI, T2-WI, and CE-T1WI and of 3 × 3 × 3 mm^3^ in DWI with a b value of 800 sec/mm^2^ and ADC maps with spline interpolation. Radiomic feature calculations were obtained using the PyRadiomics package (https://github.com/Radiomics/pyradiomics/) [25]. Within each VOI, (a) 18 first-order features, (b) 14 volume and shape features, and (c) 68 texture features were extracted as the original radiomic features. We only used original radiomic features acquired from each of the five sequences and divided them into three sets; T1 and T2 features (T features) from T1-WI and T2-WI, CE features from CE-T1WI, and diffusion and ADC features (D features) from DWIs and ADC maps. Volume and shape features were represented by those features extracted from DWI.

#### 3. Feature selection and classification model building

**Fig 2:**
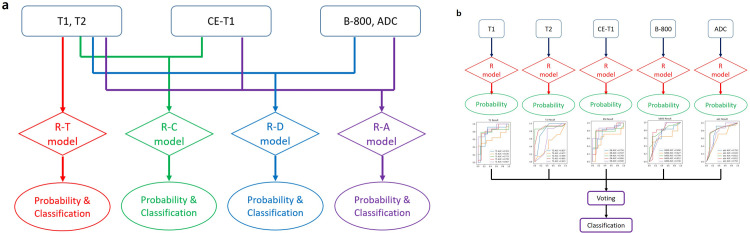
Pipeline of radiomics workflow. (a) Four alternative combinations of the three input feature sets were demonstrated in type-1 radiomics models with multi-sequence inputs. (b) Five R models built from one of five sequences containing including T1, T2, CE, diffusion, and ADC features generated type-2 radiomics model with ensemble learning approach.

We used Python to construct the classification model with the least absolute shrinkage and selection operator (Lasso) regression and R algorithm. Lasso regression is frequently used for feature selection and overfitting prevention with L1 regularization [26]. The best lambda value which minimize the misclassification error is acquired through the setting of hyperparameter in Lasso regression, and the feature importance is determined by the numerical order of the regression coefficients. The parameters for Lasso regression are summarized in the S1 Appendix. The R classifier is a well-established classifier in radiomics and is used to produce multiple decision trees [27]. R integrates votes from each decision tree composing the forest and eventually acquires a percentage of votes that favors one class. The model development was performed on personal computer equipped with 11th Gen Intel Core i9-11900K 3.50GHz processor (Intel Corporation, Santa Clara, California). Multiple classification models were developed in two ways depending on how the information in each MR sequence was incorporated. The type-1 radiomics model is a traditional method of combining features. The four combinations of three input feature sets were as follows: 1) T features only (R-T model based on T1-WI and T2-WI), 2) T + CE features (R-C model based on T1-WI, T2-WI and CE-T1WI), 3) T + D features (R-D model based on T1-WI, T2-WI, DWI and ADC maps), and 4) T + CE + D features (R-A model based on T1-WI, T2-WI, CE-T1WI, DWI and ADC maps). The type-2 radiomics model is an ensemble model using five R models that were constructed from one of five sequences using T1, T2, CE, diffusion, and ADC features. In the ensemble model, the final classification was determined by soft voting using averaged probability from five, single-sequence-based R models. R algorithm itself is also based on ensemble learning method, but it generates the machine learning model based on bootstrapping of features to make subgroups for multiple decision trees, and this process can obscure the individual importance of features from each MR sequence for differentiation of benign and malignant STTs. However, as we applied the additional ensemble learning method for incorporation of information from each MR sequence in type-2 radiomics model, it can preserve the individual importance of features from each MR sequence. The hyperparameters were manually tuned in the type-1 radiomics models (See S2 Appendix for further details) and determined via the Grid Search algorithm in each of the five models consisting of the type-2 ensemble model (See S3 and S4 Appendices for further details). All steps for all radiomics workflows are demonstrated in Fig 2.

### Statistical analysis

Student’s *t*-test and chi-square tests were used to assess the difference between benign and malignant STTs. During image preprocessing, the Dice coefficient was calculated to measure similarity between the segmentations drawn by the three readers [28]. The Scikit-learn toolboxs were used to conduct the R classifier model (sklearn_ensemble.RandomForestClassifier) and Grid Search algorithm (sklearn model_selection.GridSearchCV) in Python. To assess performance of each radiomics model, five-fold cross-validation was performed, so 125 patients were divided into 5 parts including 25 patients in each part. Then, the training and test sets were consisted of 100 and 25 patients, respectively, and the model was validated by changing test set to one of 5 parts sequentially in each fold. The average of area under the receiver operating characteristic curve (AUC), sensitivity, specificity, and accuracy were calculated with the results acquired from all five folds. Differences of AUCs among type-1 radiomics models and type-2 ensemble model were evaluated using the DeLong test. All statistical tests were performed using R version 4.0.0 (http://www.r-project.org/) and MedCalc for Windows, version 19.0 (MedCalc Software, Ostend, Belgium). A *p*-value <0.05 was considered statistically significant.

## Results

In a total of 125 patients, the numbers of benign and malignant STTs were 71 (56.8%) and 54 (43.2%), respectively. The demographic data of all included tumors are shown in Table 1. There was a significant difference in age (*p* <0.001) and sex (*p* = 0.02) between benign and malignant STTs.

**Table 1:** Demographic data of soft tissue tumors in included patients.

Soft tissue tumors	
***Benign and Borderline tumors (n = 71)***	
Histology	Schwannoma (n = 39), Tenosynovial giant cell tumor (n = 9), Desmoid type fibromatosis (n = 6), Neurofibroma (n = 4), Myxoma (n = 3), Inflammatory myofibroblastic tumor (n = 2), Intramuscular hemangioma (n = 2), Angiofibroma (n = 1), Angioleiomyoma (n = 1), Fibrohistiocytic tumor (n = 1), Granular cell tumor (n = 1), Solitary fibrous tumor (n = 1), Spiradenoma (n = 1)
Location	Neck (n = 2), Shoulder and axilla (n = 7), Upper extremity (n = 14), Hand and Wrist (n = 15), Trunk (n = 5), Hip and Groin (n = 7), Lower extremity (n = 19), Foot and Ankle (n = 2)
Age (years)	50.0±14.7 (range; 24–79)
Sex (F:M)	53: 18
***Malignant tumor (n = 54)***	
Histology	Undifferentiated pleomorphic sarcoma (n = 13), Myxofibrosarcoma (n = 10), Myxoid liposarcoma (n = 7), Malignant peripheral nerve sheath tumor (n = 5), Leiomyosarcoma (n = 4), Synovial sarcoma (n = 4), Rhabdomyosarcoma (n = 3), Dedifferentiated liposarcoma (n = 2), Alveolar soft-part sarcoma (n = 1), Angiosarcoma (n = 1), Epithelioid sarcoma (n = 1), Extraskeletal myxoid chondrosarcoma (n = 1), Malignant solitary fibrous tumor (n = 1), Pleomorphic liposarcoma (n = 1)
Location	Shoulder and axilla (n = 1), Upper extremity (n = 8), Hand and Wrist (n = 2), Trunk (n = 4), Hip and Groin (n = 9), Lower extremity (n = 28), Foot and Ankle (n = 2)
Age (years)	60.3±18.7 (range; 19–88)
Sex (F:M)	29: 25

In 20 randomly selected tumor images for evaluation of segmentation reproducibility, 7 (35%) and 13 (65%) tumors were identified as benign and malignant STTs, respectively. The average and standard deviation of the Dice coefficient between the three segmentations by the three readers were 0.92 ± 0.08 (range, 0.57–1) on T1-WI, 0.93 ± 0.06 (range, 0.68–1) on T2-WI, 0.94 ± 0.06 (range, 0.71–1) on CE-T1WI, 0.92 ± 0.08 (range, 0.67–1) on b-800 DWI, and 0.90 ± 0.07 (range, 0.62–1) on ADC map.

**Fig 3:**
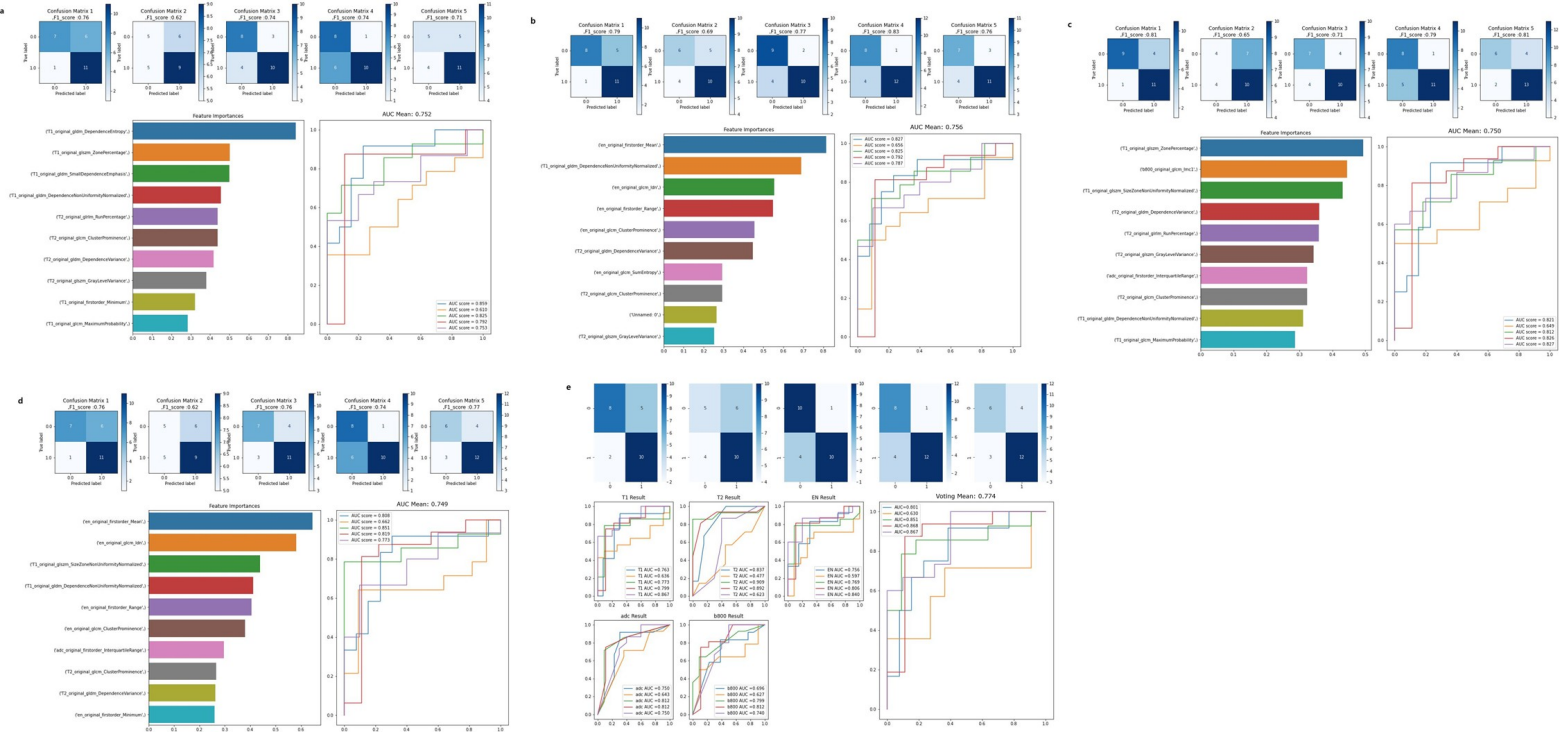
ROC curves and F1 scores of type-1 and type-2 radiomic models. (a) R-T, (b) R-C, (c) R-D, (d) R-A and (e) ensemble models showed average AUC values of 0.752, 0.756, 0.750, 0.749 and 0.774, respectively. The AUC of (e) type-2 radiomics model was superior to those of (a, b, c and d) all type-1 radiomics models, but the difference was not significant.

In the type-1 radiomics models, averaged AUC, sensitivity, specificity, and accuracy from five-fold cross validation were 0.752 (95% confidence interval, 0.667 to 0.825), 71.8%, 61.1%, and 67.2% in R-T; 0.756 (0.671 to 0.828), 76.1%, 70.4%, and 73.6% in R-C; 0.750 (0.664 to 0.823), 77.5%, 63.0%, and 71.2% in R-D; and 0.749 (0.663 to 0.822), 74.6%, 61.1%, and 68.8% in R-A models, respectively. The number of selected radiomic features through Lasso regression in each model were 11, 11, 16, and 15 in the R-T, R-C, R-D, and R-A models, respectively. Considering the feature importance, top 10 features were included for model construction and are shown in Table 2 and Fig 3. In case of models that used input features including CE features (R-C and R-A models), the number of selected CE features were predominant and showed high importance for model construction. There was no predominant feature set contributing to R model construction among T and D features. However, no apparent difference in diagnostic performance of all type-1 radiomics models was observed, even though there were variations in the number and type of input feature sets in each model construction (p-value ranged from 0.63 to 0.97 in pairwise comparison of all type-1 radiomics models). Averaged AUC, sensitivity, specificity, and accuracy of classifier ensemble construction with type-2 radiomics models were 0.774 (0.691 to 0.844), 76.1%, 68.5%, and 72.8%, respectively. The results of type-1 and type-2 radiomics models are summarized in Table 3. The ROC curves and F1 scores of each five-fold cross validation are displayed in Fig 3.

**Table 2:** Selected radiomic features after Lasso regression in the type-1 radiomics model.

	Originated sequence	Feature name
**R-T model** ^†^	**T1**	GLDM^a^_Dependence Entropy
		GLSZM^b^_Zone Percentage
		GLDM_Dependence Non Univormity Normalized
		GLCM^c^_Maximum Probability
		Shape_Elongation
		Firstorder_Minimum
	**T2**	GLDM_Dependence Variance
		GLCM_Cluster Prominence
		GLSZM_Gray Level Variance
		GLRLM^d^_Run Percentage
**R-C model** ^†^	**T1**	GLDM_Dependence Non Univormity Normalized
	**T2**	GLDM_Dependence Variance
		Firstorder_Root Mean Squared
		GLCM_Cluster Prominence
		GLSZM_Gray Level Variance
	**CE** ^**‡**^	Firstorder_Mean
		GLCM_Cluster Prominence
		Firstorder_Range
		GLCM_Idn
		GLCM_Sum Entropy
**R-D model** ^**†**^	**T1**	GLSZM_Zone Percentage
		GLDM_Dependence Entropy
		GLSZM_Size Zone Non Uniformity Normalized
		GLDM_Dependence Non Univormity Normalized
	**T2**	GLRLM_Run Percentage
		GLCM_Cluster Prominence
		GLDM_Dependence Variance
		GLSZM_Gray Level Variance
	**B-800**	GLCM_Imc1
	**ADC** ^**§**^	Firstorder_Interquartile Range
**R-A model** ^**†**^ *	**T1**	GLDM_Dependence Non Uniformity Normalized
		GLSZM_Size Zone Non Uniformity Normalized
	**T2**	GLCM_Cluster Prominence
		GLDM_Dependence Variance
	**CE** ^**‡**^	GLCM_Cluster Prominence
		Firstorder_Mean
		Firstorder_Range
		GLCM_Idn
		Firstorder_Minimum
	**ADC** ^**§**^	Firstorder_Interquartile Range

* No feature was selected in DWI

^†^ R-T, random forest model using T1+T2 features; R-C, random forest model using T1+T2+CE-T1 features; R-D, random forest model using T1+T2+DWI features; R-A, random forest model using all features

^‡^ Contrast-enhanced (CE)-T1-WI,

^§^Apparent diffusion coefficient

^a^ GLDM, Gray-level Dependence Matrix;

^b^ GLSZM, Gray-level Size Zone Matrix;

^c^ GLCM, Gray-level Co-occurrence Matrix;

^d^ GLRLM, Gray-level Run Length Matrix

**Table 3:** Diagnostic performance of the type-1 and type-2 radiomics models.

			AUC^‡^ (95% confidence interval)	p-value^§^	Sensitivity (%)	Specificity (%)	Accuracy (%)
**Radiomics models**	**Type-1**	**R-T** ^†^	0.752 (0.667–0.825)	0.32	71.8 (51/71)	61.1 (33/54)	67.2 (84/125)
		**R-C** ^†^	0.756 (0.671–0.828)	0.42	76.1 (54/71)	70.4 (38/54)	73.6 (92/125)
		**R-D** ^†^	0.750 (0.664–0.823)	0.20	77.5 (55/71)	63.0 (34/54)	71.2 (89/125)
		**R-A** ^†^	0.749 (0.663–0.822)	0.24	74.6 (53/71)	61.1 (33/54)	68.8 (86/125)
	**Type-2**		0.774 (0.691–0.844)		76.1 (54/71)	68.5 (37/54)	72.8 (91/125)

^†^ R-T, random forest model using T1+T2 features; R-C, random forest model using T1+T2+CE-T1 features; R-D, random forest model using T1+T2+DWI features; R-A, random forest model using all features

^‡^ Area under the receiver operating curve

^§^Acquired using Delong test of each type-1 model, compared to type-2 model

The AUC of a type-2 radiomics model was superior to those of all type-1 radiomics models, but the difference was not significant (p-value = 0.32, 0.42, 0.22, 0.24 compared to R-T, R-C, R-D and R-A model, respectively). In addition, the AUC of the final ensemble model in the type-2 radiomics model was higher than those of all individual models (T1WI model; AUC = 0.710 (0.622 to 0.787), T2WI model; AUC = 0.757 (0.672 to 0.829), CE-T1WI model; 0.716 (0.628 to 0.793), DWI model; AUC = 0.717 (0.629 to 0.794), and ADC model; AUC = 0.672 (0.582 to 0.753)). For further information, computational time for type-1 radiomics model (average training and test time = 34.65 and 3.40ms in R-A model) was shorter compared to type-2 radiomics model (average training and test time = 47.64 and 4.45ms in all individual models, average time for ensemble learning = 22.26ms).

## Discussion

In this study, we evaluated the effectiveness of multi-sequence MRIs with a radiomics approach for distinguishing benign and malignant STTs. The ensemble of R classifiers with combination of single-sequence inputs showed the highest AUC with a slightly increased value compared to the other single R models with serial combination of multi-sequence features for diagnosing benign and malignant STTs.

There were a few studies regarding differentiation of benign and malignant STTs using radiomics from two conventional MR sequences [11, 12, 14, 15]. The research of Wang et al. [12] presented various AUCs according to the deployment of 12 different machine learning methods with the input of pooled features of T1- and T2-WI. They found that a combination of the Lasso feature selection method and R classification achieved the highest AUC of 0.82 in validation cohort. This result is comparable with that of the R-T model (0.752, 95% CI, 0.667–0.825) presented in our study, considering that all filtered radiomic features were included in model construction in the previous study. Also, the recent study of Yue et al. [14] showed that the AUC of combined MR features (0.892) showed no significant difference with than that of fat-suppressed T2-WI features (0.883). In our study, there was no difference of diagnostic performance of the R-A model, although the types of features selected through LASSO regression were distributed across all sequences. However, our study has an important difference with previous studies, by the point that we included all essential MR sequences that have been utilized for oncology imaging and compared individual influence of single-sequence feature inputs on radiomics by generating feature-stacking model and ensemble model.

Several previous studies demonstrated the benefit of CE and DWI MRIs for STT diagnosis. However, in our study, there was only slight improvement for diagnosing benign and malignant STTs with addition of sequence features in the radiomics model. This finding is consistent with one previous study regarding the diagnostic validity of ADC-derived radiomic features, which showed no significantly additive diagnostic value to conventional ADC measurement for differentiating benign and malignant STTs [22]. Also, another study that evaluated the predictive performance of radiomics models constructed with fat-saturated T2-WI, CE-T1-WI, and both combined images for soft tissue sarcoma grading had similar high performance between T2-based models and the combined model [15]. In our study, no difference in diagnostic performance of the R-A model was apparent, although the types of features selected through Lasso regression were distributed across all sequences. Therefore, we suspect that there is no prominent additional diagnostic value by adopting radiomic features from CE-T1-WI, DWI, or ADC maps in lieu of features from T1- and T2-WI for diagnosing malignant STTs.

Through ensemble learning, we can incorporate the individual information from each MR sequence in differentiating benign and malignant STTs. This is the first study regarding ensemble learning of radiomics on STTs, but a few studies have been published on other tumors such as brain, lung, and rectal cancers [29–31]. These studies generated ensemble models with a combination of outputs from different types of classifiers such as Nearest Neighbor or Support Vector Machine learning algorithms using a pooling set of imaging features. Unlike those studies, the ensemble model of this study was developed with a combination of outputs from multiple individual R models from each MR sequence. Through this method, we focused on integrating the information of each sequence rather than integrating the information of each machine learning method. This study found that diagnostic performance of the ensemble model was slightly higher than that of conventional feature-stacking models. Even though there was no significant difference in diagnostic performance between the two types of models, an ensemble model allow greater generality of radiomics models by preserving the individual importance of features from each MR sequence.

There are a few limitations in our study. First, this study was a retrospective single-center study with a single MR scanner. Although we validated model performances using averaged values from five-fold cross validation, external validation with data sets from different institutions is regarded as an optimal way to prove generalizability. Second, we did not compare the difference of diagnostic performance between classifier algorithms, which is typically referred to as the ensemble approach in other studies. R classifier is a representative ensemble learner, so it might be inappropriate to apply to other ensemble steps; however, R classifier is usually included in top ranking classifiers that are chosen for ensemble approaches in other studies [10, 32]. Our results showed additive diagnostic value of the additional ensemble step, so we suspect that there is no limitation for assembling R classifiers in the ensemble model. Third, we did not include clinical features like age and sex in the radiomics model construction. Based on previous radiomics research studying tumor grading and survival analysis of soft tissue sarcoma [13, 15, 33], age could be an influential feature for generating and ideal radiomics model classifying malignant soft tissue tumors. However, the focus of our study was comparing the performance of radiomics models with different feature combination methods, for which inclusion of clinical features was not crucial. Fourth, there are diverse histologic subtypes in STTs, and a certain type of tumor consists of more than 50% of the benign tumors in our study. It might be difficult to find a universally effective radiomics model for malignancy differentiation, and the diagnostic performance of generated radiomics models in STT differentiation is inferior to that of radiomics models in other tumors. However, this composition reflects the real incidence in clinical practice.

## Conclusion

In this study, we compared the diagnostic performance of radiomics models with different feature assembling methods. The radiomics model constructed with pooled features extracted from multi-sequence MRI showed better performance compared to models constructed with single-sequence MRI. However, the diagnostic performance of radiomics models constructed with multi-sequence MRI showed no prominent improvement as more sequence features were assembled. Finally, when incorporating features from multiple sequences, the ensemble model showed better diagnostic performance than conventional model using pooling of combined features as a single set.

## Supporting information

S1 Table Multi-sequence MRI parameters. (DOCX)

S1 Appendix Parameters in LASSO regression. (DOCX)

S2 Appendix Hyperparameters of type-1 radiomics model using random forest classifier. (DOCX)

S3 Appendix Parameters in GridSearchCV implementation in type-2 radiomics model. (DOCX)

S4 Appendix Hyperparameter of type-2 ensemble model, determined by GridSearchCV implementation. (DOCX)

## Abbreviations

STT: Soft tissue tumor
MRI: Magnetic resonance imaging
CE: Contrast enhancement
DWI: Diffusion-weighted imaging
ADC: Apparent diffusion coefficient
R: Random forest
TSE: Turbo spin-echo
VOI: Volume of interest
Lasso: Least absolute shrinkage and selection operator
AUC: Area under the receiver operating curve
