# Supplementary material for: Ensemble learning-based radiomics with multi-sequence magnetic resonance imaging for benign and malignant soft tissue tumor differentiation
Source: PLoS One. 2023 May 31;18(5):e0286417. doi: 10.1371/journal.pone.0286417 (PMC10231763; doi:10.1371/journal.pone.0286417)
Supplement: S1 Table — (DOCX) [file pone.0286417.s001.docx]

**S1 Table. Multi-sequence MRI parameters.**

| **Parameters** | **T1-WI** | **T2-WI** | **DWI^§^**  **(single shot)** | **Post-contrast T1-WI** |
| --- | --- | --- | --- | --- |
| **Field of view** | 100-280mm | 100-280mm | 100-330mm | 100-280mm |
| **Matrix size** | 384 x 230  – 512 x 256 | 256 x 128  – 512 x 256 | 98 x 84  – 128 x 128 | 384 x 230  – 512 x 256 |
| **TR (msec)/TE (msec)^†^** | 650-750/  11-15 | 3400-4800/  68-81 | 3500-5500/  50-89 | 700-820/  15-18 |
| **Number of Slices** | 25-40 | 25-40 | 20-25 | 25-40 |
| **Slice thickness** | 3-8 | 3-8 | 3-10 | 3-8 |
| **Intersection gap** | 0-0.8 | 0-0.8 | 0-1 | 0-0.8 |
| **Turbo factor or** ^‡^**EPI factor** | 3 | 13-17 | 31-58 | 3-5 |
| **Number of excitation** | 1 | 1 | 1-8 | 1 |
| **Fat suppression** |  | STIR or Dixon |  | CHESS or Dixon |

^†^Repetition time/Echo time, ^‡^Echo planar imaging, ^§^Diffusion weighted image
