# Supplementary material for: Ensemble learning-based radiomics with multi-sequence magnetic resonance imaging for benign and malignant soft tissue tumor differentiation
Source: PLoS One. 2023 May 31;18(5):e0286417. doi: 10.1371/journal.pone.0286417 (PMC10231763; doi:10.1371/journal.pone.0286417)
Supplement: S1 Appendix — (DOCX) [file pone.0286417.s002.docx]

**S2 Appendix. Parameters in LASSO regression**

Adapted from

<https://scikit-learn.org/stable/modules/generated/sklearn.linear_model.LogisticRegression.html>

The parameters are used:

- Penalty : ‘l1’

- Dual : False

- Tol : 0.0001

- C : 1.0

- Fit_intercept : True

- Intercept_scaling : 1

- Class_weight : None

- Random_state : None

- Solver : ‘liblinear’

- Max_iter : 100

- Multi_class : ‘auto’

- Verbose : 0

- Warm_start : False

- N_jobs : None

- L1_ratio : 1
