# Supplementary material for: Ensemble learning-based radiomics with multi-sequence magnetic resonance imaging for benign and malignant soft tissue tumor differentiation
Source: PLoS One. 2023 May 31;18(5):e0286417. doi: 10.1371/journal.pone.0286417 (PMC10231763; doi:10.1371/journal.pone.0286417)
Supplement: S2 Appendix — (DOCX) [file pone.0286417.s003.docx]

**S3 Appendix. Hyperparameters of type-1 radiomics model using Random Forest Classifier**

The parameters are used:

- Number of trees in the forest: 50

- Function to measure the quality of a split: ‘gini’

- Maximum depth: 3

- Minimum number of samples required to split an internal node: 9

- Minimum number of samples required to be at a leaf node: 1

- Minimum weighted fraction of the sum of total weights required to be at a leaf node: 0

- Number of features to consider when looking for the best split: 3

- Unlimited number of leaf nodes

- Min_impurity_decrease = 0

- Min_impurity_split = 0

- Bootstrap = True

- Use out-of-bag samples to estimate generalization accuracy: False
