# Supplementary material for: Ensemble learning-based radiomics with multi-sequence magnetic resonance imaging for benign and malignant soft tissue tumor differentiation
Source: PLoS One. 2023 May 31;18(5):e0286417. doi: 10.1371/journal.pone.0286417 (PMC10231763; doi:10.1371/journal.pone.0286417)
Supplement: S3 Appendix — (DOCX) [file pone.0286417.s004.docx]

**S4 Appendix. Parameters in GridSearchCV implementation in type-2 radiomics model**

Adapted from

[https://scikit-learn.org/stable/modules/generated/sklearn.model_selection.GridSearchCV.html?highlight=gridsearch#sklearn.model_se[…]ion.GridSearchCV](https://scikit-learn.org/stable/modules/generated/sklearn.model_selection.GridSearchCV.html?highlight=gridsearch#sklearn.model_selection.GridSearchCV)

The parameters are used:

- Estimator : RandomForestClassifier

- Param_grid : n_estimators range (50, 60), max_depth range (1, 10), max_sample range (1,100)

- Scoring : None

- N-jobs : None

- Refit : True

- Cv : None

- Verbose : 0

- Pre_dispatch : n_jobs

- Error_score : np.nan

- Return_train_score : False
