# Supplementary material for: Ensemble learning-based radiomics with multi-sequence magnetic resonance imaging for benign and malignant soft tissue tumor differentiation
Source: PLoS One. 2023 May 31;18(5):e0286417. doi: 10.1371/journal.pone.0286417 (PMC10231763; doi:10.1371/journal.pone.0286417)
Supplement: S4 Appendix — (DOCX) [file pone.0286417.s005.docx]

**S5 Appendix. Hyperparameter of type-2 ensemble model, determined by GridSearchCV implementation**

The parameters are used:

1) Model constructed with T1 features

- Number of trees in the forest: 59

- Function to measure the quality of a split: ‘gini’

- Maximum depth: 1

- Minimum number of samples required to split an internal node: 2

- Minimum number of samples required to be at a leaf node: 1

- Minimum weighted fraction of the sum of total weights required to be at a leaf node: 0

- Number of features to consider when looking for the best split: 11

- Unlimited number of leaf nodes

- Min_impurity_decrease = 0

- Min_impurity_split = 0

- Bootstrap = True

- Use out-of-bag samples to estimate generalization accuracy: False

2) Model constructed with T2 features

- Number of trees in the forest: 7

- Function to measure the quality of a split: ‘gini’

- Maximum depth: 7

- Minimum number of samples required to split an internal node: 2

- Minimum number of samples required to be at a leaf node: 1

- Minimum weighted fraction of the sum of total weights required to be at a leaf node: 0

- Number of features to consider when looking for the best split: 98

- Unlimited number of leaf nodes

- Min_impurity_decrease = 0

- Min_impurity_split = 0

- Bootstrap = True

- Use out-of-bag samples to estimate generalization accuracy: False

3) Model constructed with CE features

- Number of trees in the forest: 16

- Function to measure the quality of a split: ‘gini’

- Maximum depth: 2

- Minimum number of samples required to split an internal node: 2

- Minimum number of samples required to be at a leaf node: 1

- Minimum weighted fraction of the sum of total weights required to be at a leaf node: 0

- Number of features to consider when looking for the best split: 15

- Unlimited number of leaf nodes

- Min_impurity_decrease = 0

- Min_impurity_split = 0

- Bootstrap = True

- Use out-of-bag samples to estimate generalization accuracy: False

4) Model constructed with diffusion features

- Number of trees in the forest: 9

- Function to measure the quality of a split: ‘gini’

- Maximum depth: 1

- Minimum number of samples required to split an internal node: 2

- Minimum number of samples required to be at a leaf node: 1

- Minimum weighted fraction of the sum of total weights required to be at a leaf node: 0

- Number of features to consider when looking for the best split: 29

- Unlimited number of leaf nodes

- Min_impurity_decrease = 0

- Min_impurity_split = 0

- Bootstrap = True

- Use out-of-bag samples to estimate generalization accuracy: False

5) Model constructed with ADC features

- Number of trees in the forest: 4

- Function to measure the quality of a split: ‘gini’

- Maximum depth: 1

- Minimum number of samples required to split an internal node: 2

- Minimum number of samples required to be at a leaf node: 1

- Minimum weighted fraction of the sum of total weights required to be at a leaf node: 0

- Number of features to consider when looking for the best split: 16

- Unlimited number of leaf nodes

- Min_impurity_decrease = 0

- Min_impurity_split = 0

- Bootstrap = True

- Use out-of-bag samples to estimate generalization accuracy: False
